# Electrospun Membrane Surface Modification by Sonocoating with HA and ZnO:Ag Nanoparticles—Characterization and Evaluation of Osteoblasts and Bacterial Cell Behavior In Vitro

**DOI:** 10.3390/cells11091582

**Published:** 2022-05-08

**Authors:** Julia Higuchi, Katarzyna Klimek, Jacek Wojnarowicz, Agnieszka Opalińska, Agnieszka Chodara, Urszula Szałaj, Sylwia Dąbrowska, Damian Fudala, Grazyna Ginalska

**Affiliations:** 1 Laboratory of Nanostructures, Institute of High Pressure Physics, Polish Academy of Sciences, 01-142 Warsaw, Poland; jacek.wojnarowicz@tlen.pl (J.W.); a.opalinska@labnano.pl (A.O.); achodara@leyton.com (A.C.); u.szalaj@labnano.pl (U.S.); sdabrowska@leyton.com (S.D.); d.fudala@labnano.pl (D.F.); 2 Chair and Department of Biochemistry and Biotechnology, Faculty of Pharmacy, Medical University of Lublin, 20-093 Lublin, Poland; katarzyna.klimek@umlub.pl (K.K.); g.ginalska@umlub.pl (G.G.); 3 Faculty of Materials Science and Engineering, Warsaw University of Technology, 02-507 Warsaw, Poland

**Keywords:** regenerative medicine, bone regeneration, electrospinning, sonocoating, nanohydroxyapatite, bimetallic nanocomposites, cellular responses

## Abstract

Guided tissue regeneration and guided bone regeneration membranes are some of the most common products used for bone regeneration in periodontal dentistry. The main disadvantage of commercially available membranes is their lack of bone cell stimulation and easy bacterial colonization. The aim of this work was to design and fabricate a new membrane construct composed of electrospun poly (D,L-lactic acid)/poly (lactic-co-glycolic acid) fibers sonocoated with layers of nanoparticles with specific properties, i.e., hydroxyapatite and bimetallic nanocomposite of zinc oxide–silver. Thus, within this study, four different variants of biomaterials were evaluated, namely: poly (D,L-lactic acid)/poly (lactic-co-glycolic acid) biomaterial, poly(D,L-lactic acid)/poly (lactic-co-glycolic acid)/nano hydroxyapatite biomaterial, poly (D,L-lactic acid)/poly (lactic-co-glycolic acid)/nano zinc oxide–silver biomaterial, and poly (D,L-lactic acid)/poly (lactic-co-glycolic acid)/nano hydroxyapatite/nano zinc oxide–silver biomaterial. First, it was demonstrated that the wettability of biomaterials—a prerequisite property important for ensuring desired biological response—was highly increased after the sonocoating process. Moreover, it was indicated that biomaterials composed of poly (D,L-lactic acid)/poly (lactic-co-glycolic acid) with or without a nano hydroxyapatite layer allowed proper osteoblast growth and proliferation, but did not have antibacterial properties. Addition of a nano zinc oxide–silver layer to the biomaterial inhibited growth of bacterial cells around the membrane, but at the same time induced very high cytotoxicity towards osteoblasts. Most importantly, enrichment of this biomaterial with a supplementary underlayer of nano hydroxyapatite allowed for the preservation of antibacterial properties and also a decrease in the cytotoxicity towards bone cells, associated with the presence of a nano zinc oxide–silver layer. Thus, the final structure of the composite poly (D,L-lactic acid)/poly (lactic-co-glycolic acid)/nano hydroxyapatite/nano zinc oxide–silver seems to be a promising construct for tissue engineering products, especially guided tissue regeneration/guided bone regeneration membranes. Nevertheless, additional research is needed in order to improve the developed construct, which will simultaneously protect the biomaterial from bacterial colonization and enhance the bone regeneration properties.

## 1. Introduction

One of the most serious problems in dentistry is insufficient bone volume to support teeth in the jaw [1]. Guided tissue regeneration (GTR) and guided bone regeneration (GBR) are surgical procedures that aim to restore missing bone and are commonly used to solve tissue defects in dentistry [2,3]. GTR and GBR are based on the principle of regenerating lost structures through differential tissue responses using barrier membranes. Membranes’ aim is to direct the growth of new bone tissue while preventing growth of epithelial tissue into the defect. In the last few decades, a variety of non-resorbable and resorbable barrier membranes have been developed for this purpose [2,4,5,6]. The membrane must have an optimal porous structure for the growth of cells and the exchange of nutrients [7]. One of the facile methods of fabricating porous membranes is electrospinning from polymer solutions [8]. The electrospun fibers provide good attachment for the cells and nutrient supply to the tissues [9,10]. In addition, electrospun membranes can promote epithelialization and angiogenesis by stimulating the growth of the extracellular matrix (ECM). Fibers provide an excellent roadmap for cell growth and wound healing, eventually leading to a significant reduction in remaining scar tissue [11,12,13]. Nevertheless, despite the commercially available electrospun and non-electrospun barrier membranes showing certain good clinical results, they are at risk of biomaterial-centered infections [14,15,16]. During and after operation in areas such as the oral cavity, membranes are exposed to bacteria [17]. Therefore, the development of electrospun GBR/GTR membranes supporting bone regeneration with sufficient antibacterial function may be the answer for such a challenge [18,19]. To minimize the microbial influence on GTR/GBR regenerative procedures, the addition of biocidal substances to membranes has been broadly investigated [20,21,22,23]. The biological performance of membranes was shown to be improved by the introduction of antibacterial nanoparticles [24,25]. Recent studies have shown that antimicrobial action during the bone regeneration process may have a beneficial influence on the early phases of wound healing and improve the overall clinical outcome [26].

Commercially available GTR/GBR membranes have a long history of clinical application, but they are usually not able to inhibit bacterial colonization [27]. Early membrane failure in periodontal dentistry is associated with inflammation caused by certain strains of bacteria, including streptococci, anaerobic Gram-positive cocci, and anaerobic Gram-negative rods [28]. Therefore, it is important to create new bone regeneration membranes capable of inhibiting the growth of these bacterial strains. Various antimicrobial substances, mainly antibiotics, are used to treat bacterial bone infections [29]. However, the efficiency of using membranes with antibiotics is difficult to predict due to the individual variability in patients [30]. One of the alternatives to antibiotics is the application of antibacterial-engineered nanomaterials. Due to their unique physico-chemical properties, such as a small size and high surface-to-volume ratio, they provide a broad spectrum of bacterial and fungal inhibition. Among these nanomaterials, nano zinc oxide (nZnO) is highly attractive for various applications, from solar cells, antireflection coatings, and photocatalysis to drug delivery and antibacterial coatings [31,32]. Zinc oxide is often used in bone implants because its ions take part in various metabolic processes in the body and exhibit selective toxicity towards bacteria and human cells [33]. Zinc plays an important regulatory role in human bone formation, as almost 30% of it is stored in bone tissue. In addition to being an important element of bone composition, it also activates proteins taking part in bone homeostasis. ZnO can be fabricated in microparticulate and nanoparticulate forms, and both have been shown to accelerate bone growth and mineralization [34,35,36,37]. Nanosized zinc oxide exhibits stronger antibacterial properties, which come from three aspects: surface interaction between nanoparticles and bacteria, the release of zinc ions (Zn^2+^), and the generation of reactive oxide species (ROSs) [38,39,40]. Bacteria possess negative charges on their surfaces because of the negatively charged cell wall, whereas zinc oxide nanoparticles are positively charged in aqueous suspensions [39]. The electrostatic attraction between bacteria and nZnO leads to the accumulation of nanoparticles on the surface of microbes, which changes the zeta potential of the bacteria and destroys the potassium channels of cell membranes, eventually leading to cell death [41]. To strengthen the antibacterial effect, nZnO is often combined with Ag nanoparticles (nAgs) to obtain bimetallic nanocomposites (BNCs) [42,43,44]. Among these nanocomposites, the combination of nZnO and nAgs has received much consideration because of its vast range of applications [45]. Such nanocomposites are preferred over monometallic particles due to their immensely improved optical, catalytic, and biological properties [46,47]. nAgs used to fabricate bimetallic nanocomposites are some of the most commonly used biocidal agents, with broad-spectrum antibacterial effects towards many bacterial strains [47]. Nevertheless, when used alone, nAgs can induce oxidative stress, impacting cell proliferation [48,49]. When combined with different nanoparticles (e.g., nZnO), nAgs exhibit enhanced antibacterial ability and less toxicity than with a high concentration of a single material [50]. Zhang et al. reported that coatings composed of hydroxyapatite (HA), nZnO, and nAgs on titanium implants improve osteointegration and actively prevent implant-related infection [42]. The mixture of nZnO and nAgs reduces the initial nAg content to weaken the cytotoxicity of Ag^+^ without impairing the antibacterial efficacy. Bimetallic nanocomposites (BNCs) of nZnO and nAgs synthesized in a single process (co-synthesis) may preserve the antibacterial activity of the nanoparticles while increasing the biocompatibility of the resultant composite structure [51,52]. Thus, for this study we used microwave synthesis to fabricate the well-dispersed nZnO:Ag composite at the nanoscale [32].

HA is another common material used for GTR/GBR membranes and is often used in the design of bone-contacting materials due to good bioactivity, improved bone cell growth (osteoconduction), stable anchoring to native bone (osteointegration), stimulation of host cells to develop into the osteogenic lineage (osteoinduction), and increased vascularity. The role of HA in composite membranes is to support bone regeneration by stimulating bone cells to proliferate on the material [9]. In human bone, organic HA nanocrystals are attached to the inorganic collagen fibrils [53]. Thus, scientists often suggest combining nanometric HA with the fibrous structures to mimic the natural structure of bone [54]. For example, Maimaiti et al. fabricated coatings from nHA and nZnO deposited on fibers by pulse electrochemical deposition [55]. This surface modification exhibited good antibacterial properties while retaining mineralization of the implant. Shitole et al. added nanoparticles in the polymer mixture and obtained electrospun polycaprolactone/HA/ZnO membranes [56]. These materials had higher alkaline phosphatase (ALP) activity and a better mineralization capacity than pure PCL fibers. In vitro studies of these membranes have shown that they exhibit good antibacterial properties at 10 wt% of nZnO and no cytotoxic effect towards MG-63 osteoblastic cells. Gnaneshwar et al. used nZnO/HA particles incorporated into PLA-co-PCL and silk fibroin fibers. They found that membranes with nZnO/HA particles exhibited stronger osteogenic effects than fibers containing the same quantities of nZnO and nHA alone [57].

One of the approaches to successfully attach nanohydroxyapatite, nZnO:Ag, and various nanocomposites to the electrospun fibers of membranes is the ultrasonic coating method [9,58,59,60]. Ultrasonic coating, also known as sonocoating, is based on the application of high-intensity ultrasound for the deposition of thin layers of particles on porous surfaces [61]. Our research team presented GTR/GBR membranes coated with two subsequent layers of nHA particles to obtain the stable release of calcium ions, stimulating bone regeneration [9]. Such structures composed of electrospun fibers homogenously coated with nHA proved to be favorable for the proliferation of MG-63 osteosarcoma cells on the surface.

The aim of the current study was to investigate whether applying two subsequent layers of nanoparticles characterized by osteoconductive (nHA) and antibacterial bimetallic nanocomposite (nZnO:Ag) properties on a polymer electrospun structure can support inhibition of bacterial growth without causing major toxic effects towards osteoblastic cells. To fabricate the electrospun structure, poly (D,L-lactic acid) (PDLLA) and poly(lactic-co-glycolic acid) (PLGA) polymers were mixed at a 50:50 ratio. The rationale for using PDLLA and PLGA in membrane preparation was their excellent processability in electrospinning, degradation time tailored for GTR/GBR membranes, and good mechanical and biological properties [5,9]. To investigate the bacterial inhibition of the fabricated membranes, Gram-positive and Gram-negative bacterial strains were used. To study the potential cytotoxic effect of the nanoparticles and verify the future biological environment responses, we performed in vitro culture studies with human hFOB 1.19 osteoblast cells. These cells were selected because they are reported to be a homogenous, rapidly proliferating model system for studying osteoblast differentiation and physiology and the influence of different substances on cell function [62].

## 2. Materials and Methods

### 2.1. Materials

The GMP-grade PDLLA (PURASORB^®^ PDL 20, Mw = 230 kDa) and PLGA (LA50/GA50, PURASORB^®^ PDLG 5010, Mw = 130 kDa) polymers were purchased from Corbion (Amsterdam, Netherlands). 2,2,2-Trifluoroetanol (TFE) organic solvent for electrospinning was purchased from Merck (Darmstadt, Germany). Phosphate-buffered saline (PBS, pH 7.4) was purchased from Fresenius Kabi (Bad Homburg vor der Höhe, Germany). *Staphylococcus aureus* (Gram-positive, ATCC 6538), *Escherichia coli* (Gram-negative, ATCC 8739), and normal human fetal osteoblasts (hFOB 1.19, ATCC CRL-11372^TM^) were purchased from American Type Culture Collection (ATCC). DMEM/Ham’s F12 medium, G418 disulfate salt, fetal bovine serum (FBS), penicillin–streptomycin solution, Live/dead Cell Double Staining Kit, paraformaldehyde, Triton-X100, Hoechst 33342, Mannitol Salt Agar, and MacConkey Agar were supplied by Sigma-Aldrich, St.Louis, MO, USA. AlexaFluor^TM^635 Phalloidin was purchased from ThermoFisher Scientific, Waltham, MA, USA, while fetal bovine serum (FBS) was purchased from PAN-Biotech (Aidenbach, Germany).

### 2.2. Methods

#### 2.2.1. Nanopowder Preparation

The nHA and nZnO:Ag powders were prepared via wet microwave synthesis by heating up the reagents in closed reaction vessels as described previously [63,64]. Briefly, nHA were synthesized by microwave hydrothermal synthesis (MHS) [63,64], and nZnO:Ag were synthesized by microwave solvothermal synthesis (MSS) [63,64]. After the synthesis, solutions containing nZnO:Ag were centrifuged and rinsed with deionized water prior to freeze-drying to obtain the powder product (Lyovac GT-2, SRK Systemtechnik GmbH, Sömmerda, Germany). Solutions containing nHA were freeze-dried without the centrifugation step. In previous studies, nZnO:0.1%Ag, nZnO:1%Ag, and nZnO:10%Ag particles were successfully obtained using the same method and exhibited strong antibacterial activity [64]. For this study, nZnO with 1% Ag were selected and synthesized.

#### 2.2.2. Nanopowder Characterization

Powder morphologies were studied using field-emission scanning electron microscopy (FE-SEM; Gemini FESEM, Ultra Plus, Carl Zeiss AG, Jena, Germany) with an acceleration voltage of 2–3 kV and transmission electron microscopy (TEM; FEI Talos F200X, Thermo Fisher Scientific, Waltham, MA, USA) with a beam energy of 2 keV. An energy-dispersive X-ray (EDX, Quantax 400, Bruker, Billerica, MA, USA) detector attached to the transmission electron microscope was used to identify the distribution of chemical elements in nanoparticles at high magnification. SEM samples were prepared for imaging by distributing a thin layer of particles on carbon tape and sputtering with gold–palladium. TEM samples were prepared by placing a droplet of nanoparticles ultrasonically distributed in ethanol on carbon-coated copper grids and drying in desiccator. Six individual SEM and HRTEM (high-resolution TEM) images were taken for each sample.

The density of the powder samples was determined in skeleton density analysis using a gas pycnometer (AccuPyc II 1340, FoamPyc V1.06, Micromeritics^®^, Norcross, GA USA). The specific surface areas (SSAs) of the powders were studied using the Brunauer–Emmett–Teller (BET) method on an SSA analyzer (Gemini 2360, V 2.01, Micromeritics^®^, Norcross, GA, USA). The average size of the nanoparticles was calculated from the Sauter mean diameter equation based on the results obtained for density and SSA, assuming that the samples contained only identical spherical particles (Equation (1)) [65].

(1) $$Sauter\;mean\;diameter\;\left(SMD\right)=\frac{A}{SSA\cdot 10^{18\;}\cdot \rho \cdot 10^{-21\;}}\;\left(nm\right)$$

where SMD is the Sauter mean diameter of the nanoparticle in nm, A is the shape factor equal to 6 for the sphere, SSA is in m^2^/g, and ρ is the density in g/cm^3^.

#### 2.2.3. Electrospinning of Membranes

**Figure 1:**
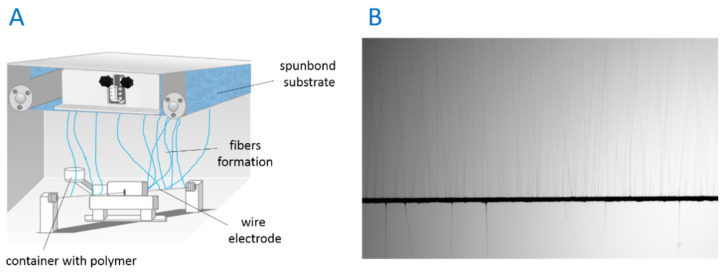
Needleless electrospinning device (**A**). The fiber formation process on the wire electrode (**B**).

PDLLA and PLGA polymer granulates were mixed in a 50/50 ratio and dissolved in the TFE solvent to obtain a solution of 20% by stirring using a magnetic stirrer in a 50 mL glass bottle with a cap for 24 h at ambient temperature. For the electrospinning process, an ISO class 8 needleless device located in the cleanroom was used (NS LAB, Elmarco, Liberec, Czech Republic) (Figure 1). The electrospinning process was conducted at a voltage of 40 kV, a distance between the wire electrode and the collector of 25 cm, polymer carriage speed of 35 cm/s, and deposition time of 30 min. Fibers were collected on the nonwoven spunbond fabric substrate and, after the electrospinning process, placed in a laminar flow cabinet in the cleanroom overnight.

#### 2.2.4. Ultrasonic Coating

PDLLA/PLGA membranes were cut into 50 mm × 50 mm square shapes. Samples were rinsed with distilled water and left for 2 h under the hood. Aqueous solutions of nHA and nZnO:Ag were prepared at 0.5% *w*/*v* for the sonocoating procedure according to a patented method [61]. PDLLA/PLGA membranes prepared for coating were mounted in the clasps and placed in a chamber equipped with a water-cooling system. A rectangular ultrasonic horn (5 cm × 20 cm) was fixed above the samples, and the chamber filled with nanoparticle solution for the designed coating layer (nHA, nZnO:Ag). The coating process was carried out over 5 min at an ultrasonic frequency of 18 kHz (±2 kHz) and temperature of 28 °C (±2 °C) to obtain uniform deposition of nanoparticles on each fiber. To form dual layers, the process was repeated for the second solution with the same parameters. Coated samples were rinsed with water and dried overnight under the hood for further analysis. The following membrane samples were obtained: PDLLA/PLGA/nHA, PDLLA/PLGA/nZnO:Ag, and PDLLA/PLGA/nHA/nZnO:Ag.

#### 2.2.5. Chemical Analysis

The chemical structures of materials were analyzed by Fourier transform infrared spectroscopy (FTIR; Tensor 27, Bruker, Billerica, MA, USA) equipped with attenuated total reflectance (Platinum ATR-Einheit A 255, Bruker, Billerica, MA, USA). The FTIR spectra were recorded by scanning the samples in the frequency range of 4000–400 cm^−1^ (16 scans for each sample at a resolution of 4 cm^–1^). The phase composition of the membranes was examined at room temperature using an X-ray diffractometer (X’Pert PRO, PANalytical, Malvern Panalytical Ltd., Malvern, United Kingdom) equipped with a Cu Kα1 anode and PIXcel1D detector. Data were collected in the range of 2θ between 10° and 100° with a scanning rate step of 3°/min. JCPDS X-ray diffraction standards data for HA, Ag, and ZnO were used to identify phases in sample X-ray diffraction patterns.

#### 2.2.6. Membrane Imaging

The Gemini FESEM (Ultra Plus, Carl Zeiss AG, Jena, Germany) with acceleration voltage of 2–3 kV was used to characterize the morphology of the membranes. Prior to imaging, the materials were coated with a nanometric gold–palladium (80/20) layer to provide conductive surfaces (Sputter Coater SCD 005/CEA 035, BAL-TEC, Balzers, Lichtenstein).

#### 2.2.7. Water Contact Angle Measurement

To evaluate the surface hydrophilicity of the membranes, static water contact angle experiments were conducted using a contact angle goniometer (DSA25B, Krüss, Hamburg, Germany). The wettability was tested using double-deionized water with a conductivity of 0.09 μS/cm according to standard PN-EN ISO3696:1999 (HLP20 UV, Hydrolab, Straszyn, Poland). Analyses were performed by observing the shape of liquid droplets placed on the sample and directly measuring the angle at the point of contact with the surface. Measurements were repeated three times for each sample type in air under controlled conditions (25 °C, 45% humidity) using a 5 mL glass syringe equipped with a flat-tip needle. Droplets (5 μL volume) were placed on the surface every 10 s (10 droplets per sample). The shape of the droplets was recorded directly using a digital camera and processed using the Krüss ADVANCE computer program (Krüss, Hamburg, Germany).

#### 2.2.8. Antimicrobial Assessment

The inhibitory activity of membranes was assessed by measuring the inhibition zone diameter of the materials incubated with bacterial strains. The antibacterial activity of PDLLA/PLGA, PDLLA/PLGA/nHA, PDLLA/PLGA/nZnO:Ag, and PDLLA/PLGA/nHA/nZnO:Ag electrospun fiber mats was determined on *S. aureus* and *E. coli* strains. Sterile bacterial test strains were streaked on solidified agar plates to attain uniform growth (Mannitol Salt Agar for *S. aureus* and MacConkey Agar for *E. coli*). Test materials in the form of 6 mm-diameter discs were then placed on the plates. Substrates with the applied test materials were incubated for 24 h at 36 ± 2 °C. After incubation, microbial growth inhibition zones were determined by optical microscopy analysis.

#### 2.2.9. In Vitro Cell Culture

The hFOB 1.19 cells were grown in DMEM/Ham’s F12 medium with the addition of 300 µg/mL G418, supplemented with 10% FBS, 100 U/mL penicillin, and 100 µg/mL streptomycin. According to ATCC recommendations, the osteoblasts were cultured at 34 °C in a humidified atmosphere with 5% CO_2_. Before cell culture studies, the membrane samples were sterilized in the wells of polystyrene plates by E-beam radiation at 25 kGy.

##### Evaluation of Cell Viability

The normal human fetal osteoblasts were seeded directly on the surface of investigated biomaterials (PDLLA/PLGA, PDLLA/PLGA/nHA, PDLLA/PLGA/nZnO:Ag, and PDLLA/PLGA/nHA/nZnO:Ag) at the concentration of 1 × 10^5^ cells/sample. After 24 h of incubation, the cell viability was assessed using a Live/Dead cell double-staining kit (Sigma-Aldrich, St.Louis, MO, USA). Immediately afterwards, the osteoblasts were observed under a confocal laser scanning microscope (Olympus Fluoview FV1000, Tokyo, Japan). The live or dead cells gave green or red fluorescence, respectively.

##### Evaluation of Cell Proliferation

The hFOB 1.19 cells were seeded on the surface of investigated biomaterials (PDLLA/PLGA, PDLLA/PLGA/nHA, PDLLA/PLGA/nZnO:Ag, and PDLLA/PLGA/nHA/nZnO:Ag) at a concentration of 5 × 10^4^ cells/sample. After 3 and 5 days of incubation, the cells were fixed with a 3.7% solution of paraformaldehyde (Sigma-Aldrich, St.Louis, MO, USA) and permeabilized with 0.2% Triton-X100 (Triton™ X-100, Sigma Aldrich, St.Louis, MO, USA). Later, the samples were stained with Hoechst solution (Hoechst 33342, Sigma-Aldrich, St. Louis, MO, USA) to visualize the cell nuclei and phalloidin-staining solution (AlexaFluor^TM^635 Phalloidin, ThermoFisher Scientific, Waltham, MA, USA) to show cytoskeletal filaments. The osteoblasts were observed under a confocal laser scanning microscope (Olympus Fluoview FV1000, Tokyo, Japan). The nuclei emitted blue fluorescence, whereas cytoskeleton filaments emitted red fluorescence. The number of cells was determined by counting nuclei in each sample.

#### 2.2.10. Statistical Analysis

The statistical differences in bacterial inhibition zones were determined using one-way ANOVA followed by Šídák’s multiple comparison test (GraphPad Prism 5, Version 5.04, Graph Pad Software Inc., San Diego, CA, USA). For evaluation of osteoblast proliferation, six independent images were taken for each biomaterial and analyzed for the purpose of counting cell nuclei using ImageJ 1.52 v software (National Institutes of Health, Bethesda, MD, USA); then, statistical analysis was performed using one-way ANOVA followed by Tukey’s multiple comparisons.

## 3. Results

### 3.1. Nanopowder Morphology

**Figure 2:**
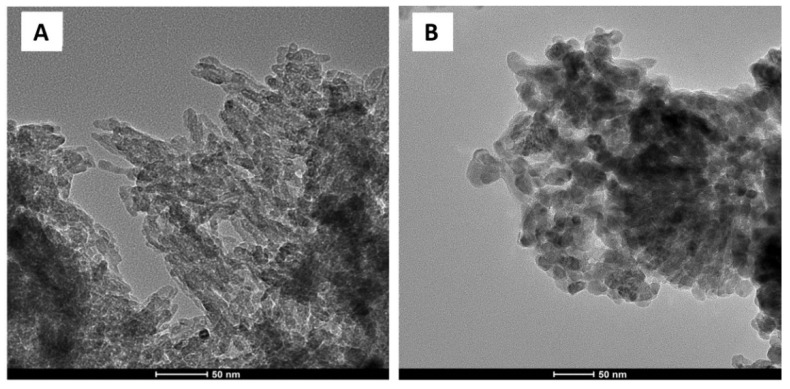
HRTEM images of nHA (**A**) and nZnO:Ag (**B**).

**Figure 3:**
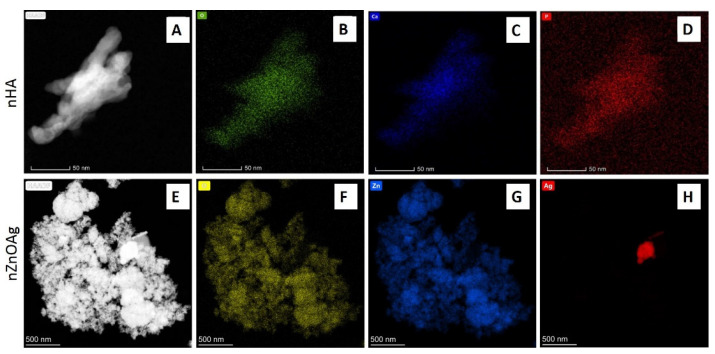
TEM images with energy-dispersive X-ray (EDX) mapping of different elements for nHA (**A**–**D**) and nZnO:Ag powders (**E**–**H**). For nHA TEM (top row), colors and complementary chemical elements on the map are as follows: (O—oxygen) green, (Ca—calcium) blue, (P—phosphorus) red. Respectively, for nZnO:Ag (bottom row), colors are: (O—oxygen) yellow, (Zn—zinc) blue and (Ag—silver) red.

Homogenous nanopowders were obtained from synthesis and freeze-drying. The TEM, BET, and density analysis demonstrated that both nHA and nZnO:Ag are pure-phase nanomaterials with a narrow distribution of parameters (Table 1). HA (GoHAP type 3) exhibited a hexagonal structure of particles in the shape of nanorods/nanoplates (Figure 2A and Figure 3). However, nZnO:Ag was characterized by more spherical morphology than the majority of nZnO (average crystallite size calculated using Scherrer’s formula (av. d) = 39 nm) and slightly bigger nAgs (av. d = 47 nm) (Figure 2B and Figure 3). The nZnO:Ag sample obtained by co-synthesis was characterized by a nZnO content of 99 molar % and 1 molar % of nAgs, which corresponds to a sample weight composition of 98.68% for ZnO and 1.32% for Ag.

### 3.2. Membrane Morphology

**Figure 4:**
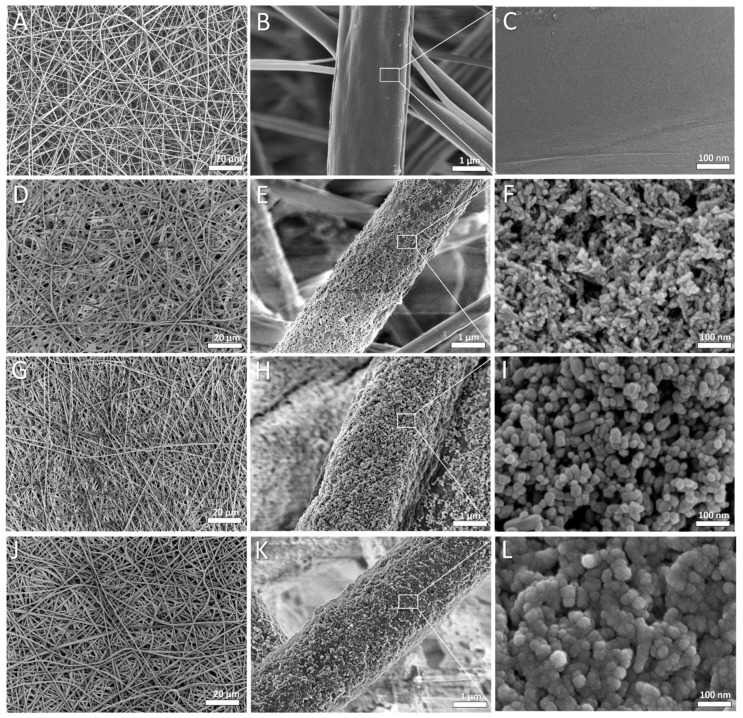
SEM images of obtained fibers: PDLLA/PLGA (**A**–**C**), PDLLA/PLGA/nHA (**D**–**F**), PDLLA/PLGA/nZnO:Ag (**G**–**I**), and PDLLA/PLGA/nHA/nZnO:Ag (**J**–**L**).

The FE-SEM images revealed that the electrospun membranes were composed of randomly distributed fibers with an average thickness of 2.08 ± 0.28 µm. The ultrasonically coated membranes were characterized by uniform coverage with nanoparticles (Figure 4). Coating with nHA particles resulted in fibers with a mean diameter of 2.22 ± 0.37 µm. The samples coated with nHA followed by nZnO:Ag had a mean diameter of 2.54 ± 0.25 µm, which indicates gradual deposition of nanoparticles on the surface.

### 3.3. Chemical Structure

**Figure 5:**
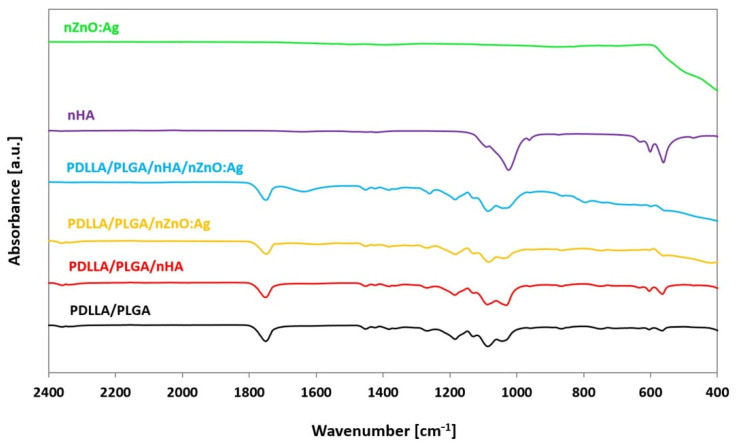
FTIR spectra of synthesized nanopowders (nHA, nZnO:Ag, electrospun polymer blend), PDLLA/PLGA, and sonocoated samples (PDLLA/PLGA/nHA, PDLLA/PLGA/nZnO:Ag, and PDLLA/PLGA/nHA/ZnO:Ag).

**Figure 6:**
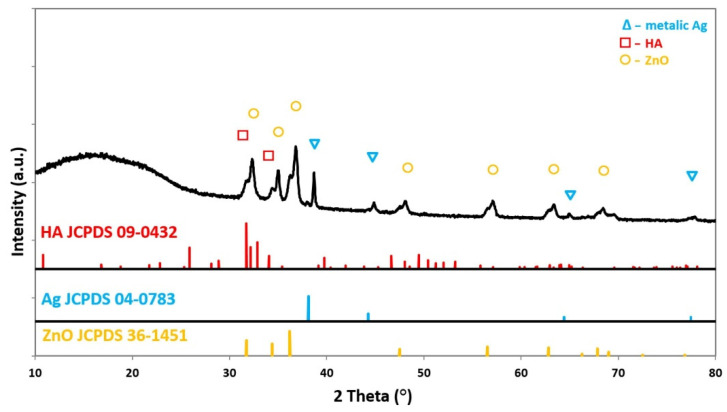
X-ray diffraction patterns of the PDLLA/PLGA/nHA/nZnO:Ag sample correlated with JCPDS data for HA, Ag, and ZnO.

Analysis of the chemical structure of coated samples by ATR-FTIR revealed the presence of peaks typical for nHA and nZnO:Ag. The results were compared to the spectra of nHA and nZnO:Ag as synthesized powders, as well as neat polymer membranes without coatings (Figure 5). The X-ray diffraction patterns of membranes coated with two subsequent layers of nHA and nZnO:Ag revealed peaks associated with HA, Ag, and ZnO (Figure 6). The composite sample had peaks positioned at 2θ values, matching the JCPDS (International Centre for Diffraction Data) data for the double nHA and nZnO:Ag coating components(HA 09-0432, Ag 04-0783, ZnO 36-1451).

### 3.4. Membrane Wettability

**Figure 7:**
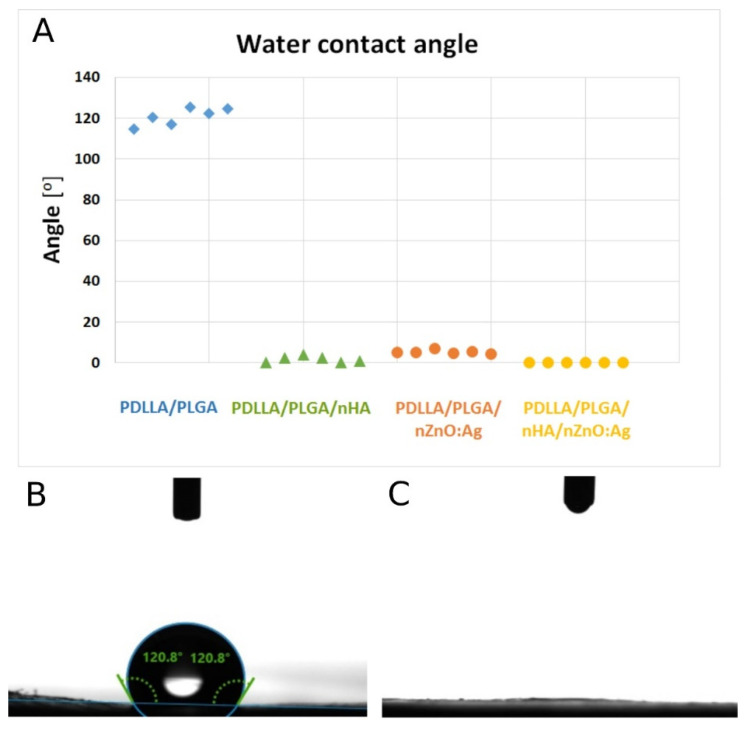
(**A**) The graph of water contact angles for the samples. (**B**) Digital camera image of water drop after 1 s on PDLLA/PLGA. (**C**) Digital camera image of water drop after 1 s on PDLLA/PLGA/nHA/nZnO:Ag (**C**).

The mean water contact angle measurements on samples (n = 6) were 120.8° (SD ± 4.26°) for PDLLA/PLGA, 5.4 (SD ± 1.02°) for PDLLA/PLGA/nZnO:Ag, 1.6 (SD ± 1.57°) for PDLLA/PLGA/nHA, and 0 (SD ± 0°) for PDLLA/PLGA/nHA/nZnO:Ag (Figure 7A). The surface wettability of polymer membranes was drastically changed from hydrophobic (Figure 7B) to highly hydrophilic (Figure 7C) after the sonocoating process. This effect was observed for all coated samples.

### 3.5. In Vitro Antibacterial Activity

**Figure 8:**
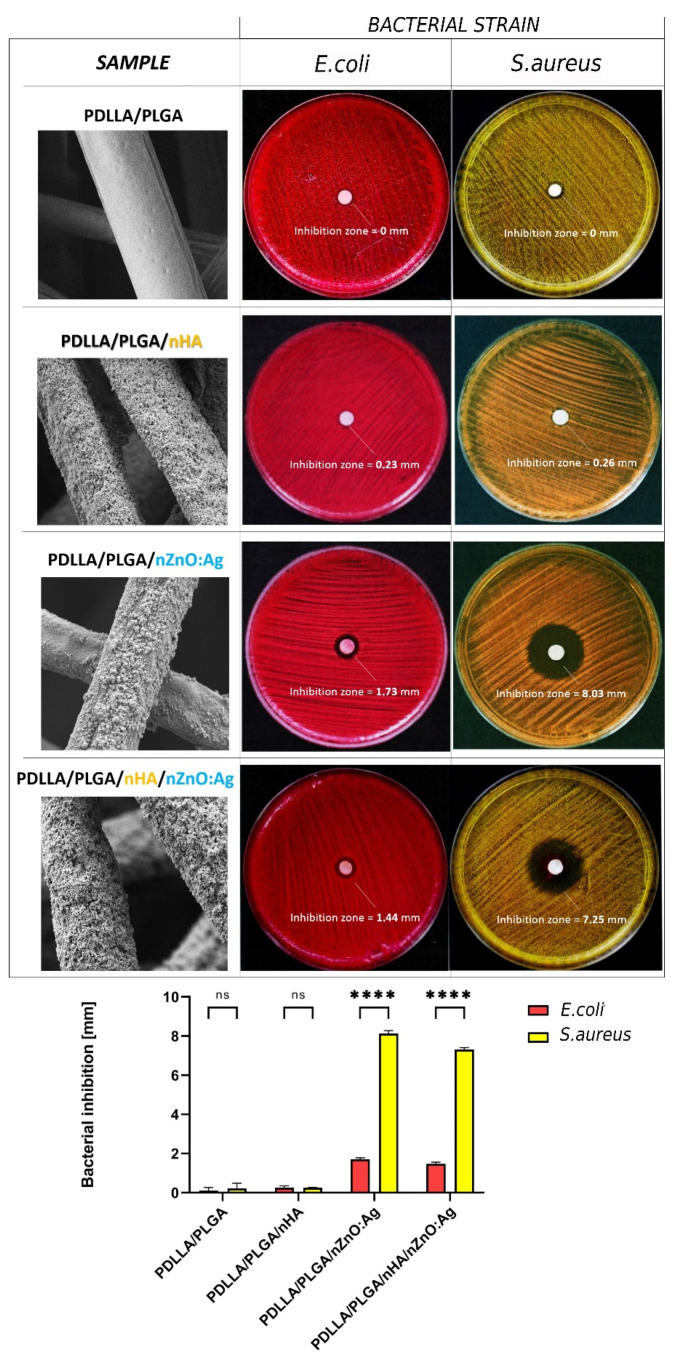
Representative SEM images and photographs of the bacterial inhibition zones formed by the different samples in contact with *Escherichia coli* and *Staphylococcus aureus* bacterial strains. The data are represented as mean ± SD (*n* = 3). Differences between tested samples were considered as statistically significant if **** *p* < 0.05, while nonsignificant results are marked as ns (one-way ANOVA, followed by Šídák’s multiple comparisons test).

The in vitro antibacterial activity of membranes against *S. aureus* and *E. coli* is shown in Figure 8. Only membranes containing nZnO:Ag were effective against both bacterial strains. This effect was more pronounced for *S. aureus* than *E. coli*. The samples containing both nZnO:Ag and nHA exhibited slightly less bacterial inhibition but were within a similar range to that of nZnO:Ag-coated samples.

### 3.6. In Vitro Osteoblast (hFOB 1.19) Viability

**Figure 9:**
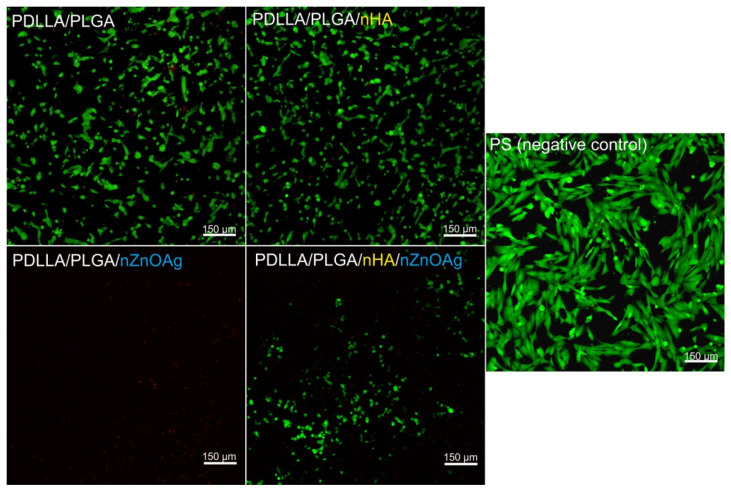
Confocal microscope images presenting the viability of normal human fetal osteoblasts (hFOB 1.19 cell line, ATCC CRL-11372TM) cultured on polystyrene (PS, negative control for cytotoxicity) and on the investigated biomaterials after 24 h of incubation. Viable cells emitted green fluorescence and dead cells red fluorescence. Magnification: 100×; scale bar: 150 μm.

After 24 h of incubation, confocal microscope observations revealed that cells grown on 2D polystyrene—PS (negative control for cytotoxicity) were viable (green fluorescence), and no dead cells were observed (red fluorescence) (Figure 9). In the case of 3D biomaterials, both PDLLA/PLGA and PDLLA/PLGA/nHA samples allowed good cellular attachment and growth (Figure 9). Similarly, to control hFOB 1.19 cells (cultured on PS), the osteoblasts grown on these biomaterials (PDLLA/PLGA and PDLLA/PLGA/nHA samples) were viable, and no dead cells were shown in the visual field. In turn, the PDLLA/PLGA/nZnO:Ag membrane exhibited an opposite effect. This scaffold did not support osteoblast growth and exhibited very high cytotoxic activity—only single cells were detected, but all of them were dead (Figure 9). In turn, cells cultured on the PDLLA/PLGA/nHA/nZnO:Ag biomaterial were alive, but their number was lower than the number of osteoblasts grown on the PS, PDLLA/PLGA, and PDLLA/PLGA/nHA scaffolds. Thus, both PDLLA/PLGA and PDLLA/PLGA/nHA biomaterials enabled proper growth of osteoblasts, while the PDLLA/PLGA/nZnO:Ag scaffold did not allow for the growth of these cells. On the other hand, the PDLLA/PLGA/nHA/nZnO:Ag biomaterial provided more a favorable template for cell growth compared to the PDLLA/PLGA/nZnO:Ag sample, which indicates that the presence of an underlayer of nHA in the PDLLA/PLGA/nHA/nZnO:Ag biomaterial reduced the cytotoxic effect of nZnO:Ag.

### 3.7. In Vitro Osteoblast (hFOB 1.19) Proliferation

**Figure 10:**
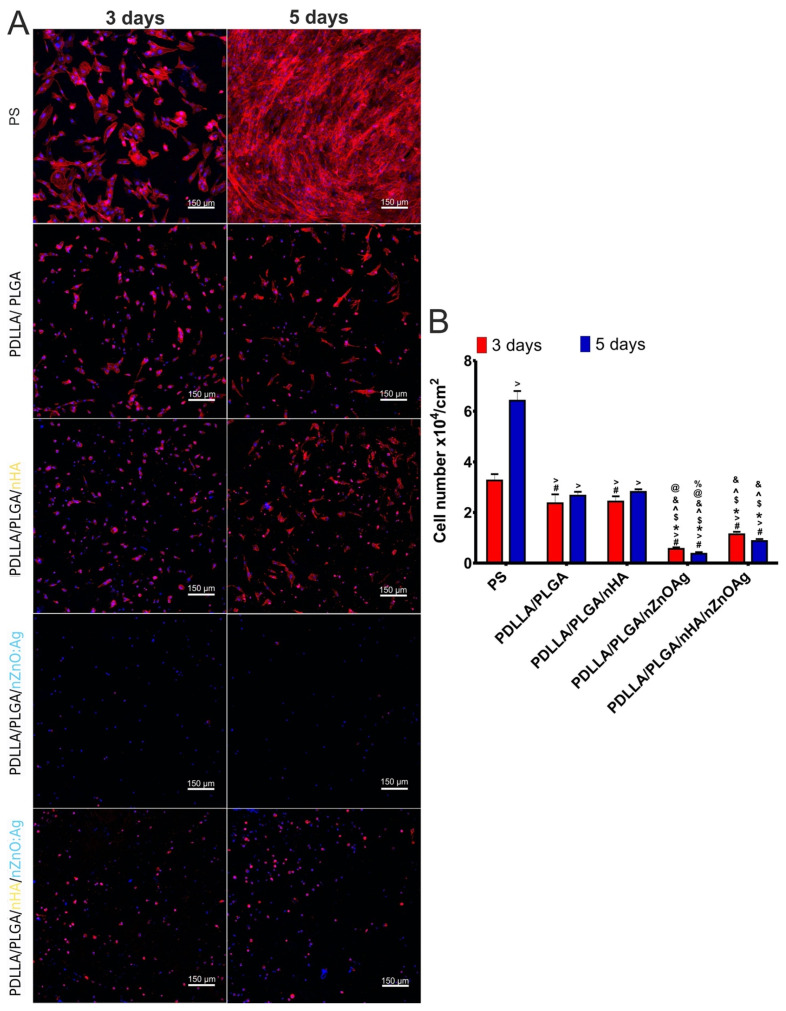
Confocal microscope images of (**A**) the morphology of normal human fetal osteoblasts (hFOB 1.19 cell line, ATCC CRL-11372^TM^) after 3 and 5 days of cell cultivation on investigated biomaterials. The cells cultured on polystyrene (PS) were considered as a control in the experiment. Nuclei emitted blue fluorescence and cytoskeleton filaments red fluorescence. Magnification: 100×; scale bar: 150 μm. (**B**) The number of cells was determined based on the obtained images. # *p* < 0.05 compared to PS at day 3; > *p* < 0.05 compared to PS at day 5; * *p* < 0.05 compared to PDLLA/PLGA at day 3; $ *p* < 0.05 compared to PDLLA/PLGA at day 5; ^ *p* < 0.05 compared to PDLLA/PLGA/nHA at day 3; & *p* < 0.05 compared to PDLLA/PLGA/nHA at day 5; @ *p* < 0.05 compared to PDLLA/PLGA/nHA/nZnO:Ag at day 3; % *p* < 0.05 compared to PDLLA/PLGA/nHA/nZnO:Ag at day 5; one-way ANOVA test followed by Tukey’s multiple comparisons.

After a 3-day incubation, confocal microscopy indicated well-attached and spread osteoblasts on the surface of polystyrene—PS (control in experiment). The cells grown on PDLLA/PLGA and PDLLA/PLGA/nHA membranes (control biomaterials) were also attached, but their cytoskeleton filaments were less extensive compared to cells cultured on PS (Figure 10A). The increase in incubation time to 5 days led to significant enhancement (*p* < 0.05) in the number of cells cultured on PS, whereas it did not cause significant changes in the number of cells cultured on PDLLA/PLGA and PDLLA/PLGA/nHA membranes (Figure 10B). However, the cells cultured on these membranes possessed a more complex system of cytoskeleton filaments (Figure 10A) compared to day 3, suggesting their good condition. It should be noted that conditions under 2D culture (on polystyrene) are completely different compared to conditions under 3D culture (on biomaterials). In other words, in many cases, the cell attachment and adaptation to the 3D environment (on biomaterials) are slower compared to these processes in the 2D environment (on polystyrene) [66,67]. Presumably, an increase in experiment time will lead to significant changes in the number of cells cultured on PDLLA/PLGA and PDLLA/PLGA/nHA membranes. In turn, the PDLLA/PLGA/nZnO:Ag biomaterial did not allow for osteoblast growth and division (Figure 10A). After 3 and 5 days of incubation, only single-cell nuclei without cytoskeleton filaments were observed, which indicates that the cells were mostly dead. Moreover, the number of cells declined with time (Figure 10B). On the other hand, the number of cells cultured on PDLLA/PLGA/nHA/nZnO:Ag was significantly higher (*p* < 0.05) than the number of osteoblasts grown on PDLLA/PLGA/nZnO:Ag, but the number also decreased with increased incubation time (Figure 10B). Moreover, these cells exhibited abnormal morphology, as they were round with very few cytoskeleton filaments. Thus, this experiment showed that PDLLA/PLGA and PDLLA/PLGA/nHA membranes enabled growth and proliferation of osteoblasts. In turn, the PDLLA/PLGA membrane enriched with nZnO:Ag did not allow for osteoblast growth and proliferation. The addition of the nHA underlayer to PDLLA/PLGA/nZnO:Ag made the surface of the biomaterial more suitable for osteoblast adhesion and growth, but the number of cells was still significantly lower compared to the number of cells cultured on PS and control biomaterials (PDLLA/PLGA and PDLLA/PLGA/nHA). Thus, these results clearly confirm the data obtained during evaluation of cell viability (Figure 9). Notably, the presented experiment was carried out under static conditions. For this reason, an in vivo study is planned to verify the in vitro results.

## 4. Discussion

**Figure 11:**
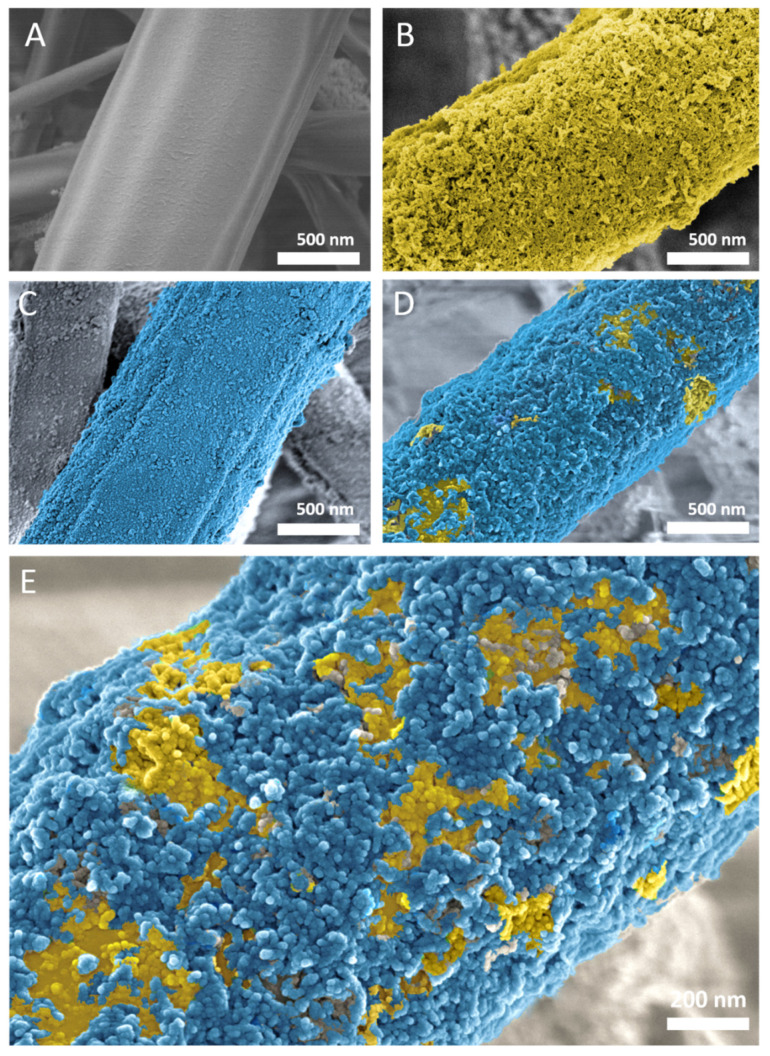
Colorized SEM image of: (**A**) non-coated PDLLA/PLGA fiber, (**B**) PDLLA/PLGA/nHA, (**C**) PDLLA/PLGA/nZnO:Ag, (**D**) PDLLA/PLGA/nHA/nZnO:Ag after coating and (**E**) PDLLA/PLGA/nHA/nZnO:Ag after 24 h immersion in PBS medium at 37 °C. Note PDLLA/PLGA/nHA/nZnO:Ag sample visible regions of nHA (yellow) and nZnO:Ag (blue).

**Figure 12:**
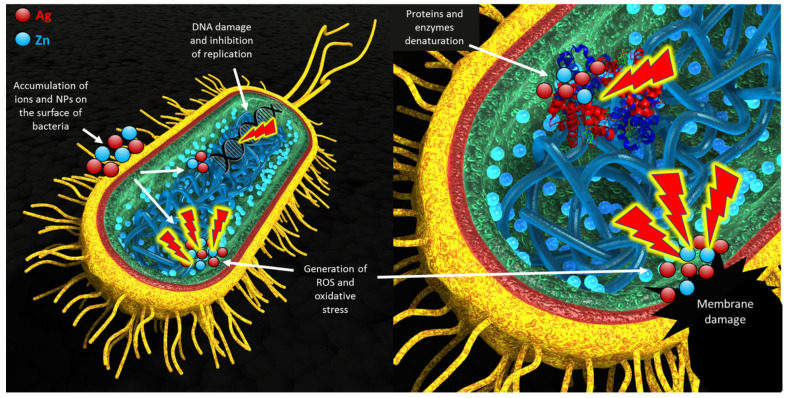
Scheme of possible bacterial cell interaction with nZnO:Ag particles released from the surface of the membrane. The figure was prepared by the first author using basic model of bacterial cell image, obtained with license type CC-BY-NC-SA 4.0.

Despite many reports on modifying electrospun fibers with nanoparticles, this study used ultrasonic coating for dual deposition of nHA and nZnO:Ag on fibers for the first time. Nanohydroxyapatite was previously used for the ultrasonic modification of implants by Higuchi et al. and Rogowska-Tylman et al. [9,60]. The rationale for using a bimetallic nanocomposite of nZnO with nAgs together with nHA was to find an equilibrium between the good antibacterial and osteogenic properties of membranes without causing a toxic effect towards bone cells. In this study, we demonstrated that the ultrasonically deposited layers composed of nanoparticles (nZnO:Ag with addition of nHA) exhibited very good antibacterial properties with a positive influence of nHA on human osteoblast cells, i.e., addition of nHA layer decreased the cytotoxicity caused by the presence of nZnO:Ag. The membranes coated with nHA and nZnO:Ag showed much higher hydrophilicity and water absorption than uncoated polymer membranes. This is associated with the hydrophilicity and high specific area of the nanoparticles used for coating, as well as the unique nanotopography formed after the coating process (Figure 11). This result is very important because the hydrophilicity of the surface plays a decisive role during the material’s first cells–biomaterial interactions in the early phases of healing and osseointegration. At the same time, the rough fibrous surface, similar to the natural ECM, supports the proliferation of osteoblasts [68]. In addition, a suitable nanotopography, hydrophilic nature, and biomolecular signals from the fibers may guide cells in their amoeboid movement [69]. Interestingly, the combination of two subsequent layers of nHA and nZnO:Ag resulted in similar bacterial inhibition as a single layer of nZnO:Ag. This proves that the presence of nHA did not jeopardize the biocidal effect of nZnO:Ag itself. Moreover, the resulting fibrous membranes with nHA and nZnO:Ag exhibited increased antibacterial activity against both Gram-negative and Gram-positive bacterial strains. Oral pathogen microbiota are composed of many bacteria [70]. However, the *S. aureus* and *E. coli* bacterial strains chosen for this study are commonly regarded as transient colonizers of the oral cavity and often disregarded when isolated from clinical specimens. In addition, several distinct oral infections (e.g., angular cheilitis, parotitis, staphylococcal mucositis) are caused by these microorganisms [71]. More recently, it has also been suggested that *S. aureus* may have a role in dental implant failure. They are both a well-established golden standard in biological studies of dental materials due to their high prevalence in oral pathogenic microbiota. Our recent work regarding nZnO:Ag coatings on titanium dental implants showed that nZnO:Ag significantly decreased the growth of not only *E. coli* and *S. aureus* but also *S. mutans* and *S. oralis* [44]. Thus, based on the above, the choice of *E. coli* and *S. aureus* as a model bacterial strain for this preliminary study seems to be justified. The bacterial inhibition effect in this study was more pronounced for *S. aureus* than *E. coli.* Although nano silver is considered by many authors as more bactericidal towards *E. coli* than *S. aureus*, it is often reported that for materials composed of nZnO and nAgs, the effect is opposite [72,73,74]. This phenomenon has also been observed and broadly described in the literature for ZnO nanoparticles with a size below 50 nm [74,75,76,77]. One of the possible mechanisms of the antibacterial effect of nZnO:Ag is due to accumulation of NPs and ions on the surface of bacteria, disruption of the cell membrane and oxidative stress (Figure 12) [78]. Furthermore, nanoparticles have stronger antimicrobial activity than their micrometric counterparts due to their small size and high surface-to-volume ratio [79]. The additional component of nAgs in the nZnO:Ag composite provided an even higher antibacterial effect than nZnO alone [42]. Therefore, the antimicrobial properties obtained in our study could be attributed to the presence of both the silver and zinc ions. Previous works proved that coatings and suspensions of co-precipitated composites were very effective against *S. aureus* and *E. coli* and exhibited good bioactivity [80]. Nevertheless, the combination of nAgs with nZnO may have a disadvantageous influence on the viability of human cells. Although Colon et al. showed that nanophase ZnO improves normal osteoblast function [81], another study indicated that toxicity related to nAgs could inhibit the cell viability [48]. Indeed, it is known that biomaterials enriched with metals possessing antibacterial properties, such as Ag, Au, Zn, and Cu, exhibit inhibitory activity towards bacterial cells, but at the same time, they are very toxic towards eukaryotic cells [82,83]. Thus, researchers are still trying to develop biomaterials that will maintain antibacterial properties without unfavorable cytotoxicity towards mammalian cells. One of the promising solutions is the addition of HA to the biomaterials enriched with metals possessing antibacterial properties. It was demonstrated that the presence of HA can neutralize the toxicity of Zn and Ag compounds. Iqbal et al. synthesized Zn–Ag–HA nanoparticles using the microwave-assisted wet precipitation process [84]. In that study, materials combined with HA exhibited excellent antibacterial properties and supported the attachment of normal human osteoblast cells (NHOst). The cell culture in this study was performed in a different cell line, human osteoblastic (hFOB 1.19) cells, which showed that the nZnO:Ag coating alone is toxic, but when combined with an nHA underlayer, it is more favorable for cells, as the cytotoxic effect is reduced. Thus, the presence of the nHA coating increased the viability and proliferation of cells on the surface of the biomaterials enriched with nZnO:Ag and nHA, when compared to biomaterial with nZnO:Ag without nHA. This phenomenon is attributed to the nanotopography and osteoblastic cell-friendly chemical nature of the nHA, which is composed of calcium and phosphorus. Woźniak et al. reported that the mechanism of nanoparticle deposition during the ultrasonic coating process is based on dendritic growth of layers [85]. We observed that, despite the uniform coverage, some regions of the nHA layer were exposed faster than the outer coating on fibers when immersed in the incubation medium for 24 h (Figure 11). Thus, it is suspected that dendritic formation of layers could contribute to simultaneous release of Zn^2+^, Ag^+^, and Ca^2+^ ions. Ca^2+^ promote growth, mineralization, and bone formation, whereas Zn^2+^ and Ag^+^ provide biocidal function.

## 5. Conclusions

In this preliminary study, we demonstrated that it is possible to tailor the cellular response and biocidal activity by applying different combinations of nanoparticulate layers on electrospun fibers. This example of precisely tailoring nanoscale architecture opens up a new field of application for this technology in tissue engineering. We obtained a unique combination that allows us to reduce cytotoxicity towards osteoblasts and maintain antibacterial properties by using the bimetallic nanocomposite nZnO:Ag and bioactive nHA as layers on a biodegradable fibrous structure. The synergy of nHA, nZnO, and nAgs enhanced the biological performance and antibacterial function of the fibers’ coating. Thus, the results of this introductory study open up a new range of possibilities of using various therapeutic nanoparticles in the form of nanometric layers on matrices for biomedical applications. Overall, the presented fibrous material enriched with nZnO:Ag and bioactive nHA may constitute a promising solution for GTR/GBR procedures in bone regeneration, but further modifications of this biomaterial are needed in order to maintain its antibacterial properties and improve the growth, proliferation, and differentiation of osteoblasts.

## 6. Patents

The Institute of High Pressure Physics PAS is an official holder of the Polish patent PL226891 (B1), “Method of manufacturing bone implant and bone implant”, granted on 29 September 2017, Polish patent PL240082B1 “Biological Barrier Membrane” granted on 17 November 2021, and the International Patent Application (WIPO/PCT) WO2020085927A1, 26.10.2019.

## Figures and Tables

**Table 1:** Parameters of the nanopowders.

Sample	Specific Surface Area by Gas Adsorption, BET (m^2^/g)	Average Size (SMD), d ± σ (nm)	Skeletal Density by Gas Pycnometry, ρ_s_ ± σ (g/cm^3^)
nHA	140	16 ± 3	2.86 ± 0.04
nZnO:Ag	33.8	39 ± 2	5.37 ± 0.01

## Data Availability

Data will be made available upon justified request.
